# A nomogram model for the occurrence of bladder spasm after TURP in patients with prostate enlargement based on serum prostacyclin and 5-hydroxytryptamine and clinical characteristics

**DOI:** 10.1590/S1677-5538.IBJU.2024.0011

**Published:** 2024-05-20

**Authors:** Pengfei Shang, Miaomiao Lan

**Affiliations:** 1 Heji Hospital Affiliated to Changzhi Medical College Department of Urology Changzhi China Department of Urology, Heji Hospital Affiliated to Changzhi Medical College, Changzhi, Shanaxi, PR. China; 2 Heping Hospital Affiliated to Changzhi Medical College Department of Obstetrics Changzhi China Department of Obstetrics, Heping Hospital Affiliated to Changzhi Medical College, Changzhi, Shanaxi, PR. China

**Keywords:** Transurethral Resection of Prostate, Prostate, prostacyclin synthetase [Supplementary Concept]

## Abstract

**Objective:**

With the development of analytical methods, mathematical models based on humoral biomarkers have become more widely used in the medical field. This study aims to investigate the risk factors associated with the occurrence of bladder spasm after transurethral resection of the prostate (TURP) in patients with prostate enlargement, and then construct a nomogram model.

**Materials and methods:**

Two hundred and forty-two patients with prostate enlargement who underwent TURP were included. Patients were divided into Spasm group (n=65) and non-spasm group (n=177) according to whether they had bladder spasm after surgery. Serum prostacyclin (PGI2) and 5-hydroxytryptamine (5-HT) levels were measured by enzyme-linked immunoassay. Univariate and multivariate logistic regression were used to analyze the risk factors.

**Results:**

Postoperative serum PGI2 and 5-HT levels were higher in patients in the Spasm group compared with the Non-spasm group (P<0.05). Preoperative anxiety, drainage tube obstruction, and elevated postoperative levels of PGI2 and 5-HT were independent risk factors for bladder spasm after TURP (P<0.05). The C-index of the model was 0.978 (0.959-0.997), with a χ2 = 4.438 (p = 0.816) for Hosmer-Lemeshow goodness-of-fit test. The ROC curve to assess the discrimination of the nomogram model showed an AUC of 0.978 (0.959-0.997).

**Conclusion:**

Preoperative anxiety, drainage tube obstruction, and elevated postoperative serum PGI2 and 5-HT levels are independent risk factors for bladder spasm after TURP. The nomogram model based on the aforementioned independent risk factors had good discrimination and predictive abilities, which may provide a high guidance value for predicting the occurrence of bladder spasm in clinical practice.

## INTRODUCTION

Prostate enlargement is a common clinical male urological disorder that is mostly observed in older population (1). The symptoms of prostate enlargement include difficult urination, urgent urination, frequent urination, nocturia, and interrupted urination, which seriously affect the quality of life of patients (2). Transurethral resection of the prostate (TURP) is a common surgical procedure for prostate enlargement with the advantages of less invasiveness and faster postoperative recovery (3). Postoperative bladder spasm is a common complication of TURP, which refers to the spastic contraction of the detrusor of the bladder, manifesting as lower urinary tract symptoms, such as urinary urgency, temporary urinary closure, and lower abdominal holding pain; in severe cases, secondary bleeding, poor drainage, and urinary tract infection (4, 5). More studies have been conducted on the factors that influence bladder spasm after TURP. However, achieving individualized predictions remains an urgent clinical challenge.

However, the pathogenesis of bladder spasms remains unclear. Studies on clinical symptoms and urodynamic findings have confirmed that detrusor instability is the most important cause of urinary frequency, urgency, and urge incontinence. Bladder detrusor instability and bladder overactivity may be influenced by neurotransmitter release or inflammatory mediators (6-8). Prostacyclin (PGI2) is an important vasoactive substance produced in bladder tissue and plays a key role in bladder homeostasis and inflammation (9). 5-Hydroxytryptamine (5-HT) is as important regulatory neurotransmitter that has been shown to be involved with the regulation of normal bladder voiding function and detrusor overactivity (10, 11).

The application of mathematical models based on humoral biomarkers in medicine has increased with the advancement of analytical techniques. Less research has been done on the use of serum markers to forecast the likelihood of bladder spasms following TURP. In this study, we investigated the factors associated with the occurrence of bladder spasm after TURP surgery by analyzing serum PGI2 and 5-HT levels, and other clinical indicators. In addition, this study established a nomogram model based on the risk factors affecting postoperative bladder spasm after TURP, and serum PGI2 and 5-HT levels to provide reference values to reduce the occurrence of postoperative bladder spasms. We hope to provide new perspectives for recognizing the incidence of postoperative bladder spasms.

## MATERIALS AND METHODS

### General Information

**Figure 1:**
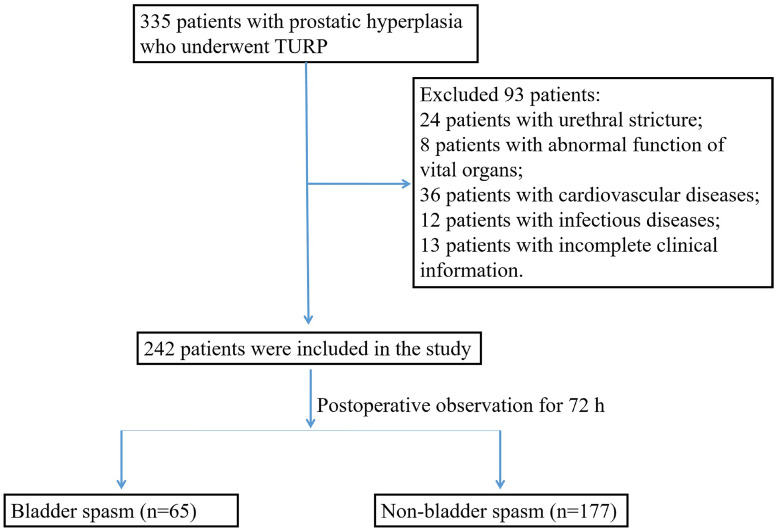
The inclusion criteria flowchart of recruited patients.

Clinical data of 242 patients with benign prostate enlargement who underwent TURP from May 2020 to August 2022 were retrospectively analyzed (Figure-1). According to the Chinese Diagnostic and Treatment Guidelines for Urological Diseases (2014), bladder spasm was diagnosed when paroxysmal pubic pain, frequent urination, and painful urination occurred within 72 h after TURP. Sixty-five patients who developed bladder spasm were in the spasm group, aged 40 to 72 years, with a mean of (53.92±12.10) years. The other 177 patients without bladder spasm were in the non-spasm group, aged 21-76 years, with a mean of (55.11±10.82) years. The inclusion criteria were prostate enlargement meeting the diagnostic criteria of the Chinese Diagnostic and Treatment Guidelines for Urological Diseases (2014), International Prostate Symptom Score (IPSS) ≥7, degree of enlargement II to III, compliance with the indications for TURP surgery, absence of abnormal postoperative vital signs, age ≥ 18 years, and complete clinical data. Exclusion criteria included contraindications to surgery, preoperative application of drugs affecting bladder function, history of pelvic surgery, history of combined bladder disease or urethral stricture, concomitant abnormal function of vital organs, cardiovascular disease, infectious disease, comorbid psychiatric disorders, or cognitive dysfunction. The study met medical ethics standards and was approved by the ethics committee of the institution (2020-CZ-006).

### Surgery

All surgeries were performed by the same surgeon. TURP was performed in all patients. All procedures were performed under spinal anesthesia by an experienced surgeon using a 24-Fr resectoscope (Richard Wolf, Germany), a bipolar generator, and saline irrigation fluid. The procedure was performed according to standard methods. Postoperatively, balloon catheters F18–F20 were left in place, and bladder irrigation was performed for 1-3 d, and patients received the same epidural self-controlled analgesia. The catheter was removed without bleeding. Bladder spasm was observed 72 h after surgery.

### Data collection

Clinical data of the patients were collected, including general information, such as age, body mass index (BMI), education, annual intake, disease duration, proportion of unstable bladder, and perioperative information. Preoperative anxiety was evaluated using the Self-rating Anxiety (SAS) (12). SAS consists of twenty questions which are scored as 1–4 points individually, resulting in a raw score of 20–80 points; subsequently the standard score is calculated by int (1.25 raw score) and ≥ 60 was considered to have anxiety. An involuntary contraction of the urethral muscle is defined as unstable bladder based on the presence of a bladder pressure ≥ 1.47 kPa during bladder filling on urodynamic examination, which cannot be actively inhibited.

### Serum indicators

Five milliliters of early morning fasting venous blood were collected from the patients preoperatively and 24 h postoperatively. After centrifugation at 3000 rpm for 10 min, serum was collected. Serum levels of PGI2 (MBS2501804; MyBioSource, California, USA) and 5-HT (SEKSM-0016; Solarbio, Beijing, China) were measured using enzyme-linked immunosorbent assay. All operations were performed according to the manufacturer's instructions. The corresponding standard curves were derived from the standard concentrations and absorbance values. Concentrations of PGI2 and 5-HT in the samples were determined using a curve equation.

### Statistical analysis

The measurement data are described as mean±SD, and two independent sample t-tests were used for comparison between groups. Count data are described as the number of cases, and comparisons between groups were performed using the χ2 test or Fisher's exact probability method. The predictive value of serum PGI2 and 5-HT levels for the occurrence of postoperative bladder spasm was assessed using a receiver operating characteristic (ROC) curve. Univariate and multivariate logistic regression models were used to screen for the risk factors for the development of postoperative bladder spasms after TURP. A nomogram model for predicting the occurrence of postoperative bladder spasm after TURP was constructed using the rms package in the R software. The bootstrap method was used to repeat sampling 100 times for internal validation. The discrimination ability of the nomogram model was assessed using the area under the ROC curve (AUC). The Hosmer-Lemeshow goodness-of-fit test, calibration curve, and decision curve analysis (DCA) were used to assess the discrimination and accuracy of the model. Differences of P<0.05 was considered statistically significant.

## RESULTS

### General Information

Bladder spasm occurred in 65 (26.86%) of the 242 patients with prostate enlargement after TURP. Age, BMI, education, yearly intake, length of illness, percentage of unstable bladder, catheter retention time, resected prostate volume, and operational time did not significantly differ between the spasm group and the non-spasticity group (P>0.05). The proportion of patients with preoperative anxiety, rinse fluid temperature <34°C, rinse fluid speed <60 or >80 drops/min, ureteral balloon injection >20 mL, drain blockage, postoperative constipation, and bladder bleeding was significantly higher in the spasm group than in the non-spasm group (P<0.05) as shown in Table-1.

**Table 1:** Clinical Characteristics of the Patients at Baseline.

Indicators	n	Non-spastic group (n=177)	Spastic group (n=65)	t/ꭓ2	P
Age (years)	177	55.11±10.82	53.92±12.10	0.734	0.464
BMI (kg/cm^2^)	65	23.69±5.39	24.95±4.42	1.694	0.092
Education				1.282	0.286
High School and below	159	120(67.80%)	39(60.00%)		
College and above	83	57(32.20%)	26(40.00%)		
Disease duration				0.056	0.872
≥3 years	174	128(72.32%)	46(70.77%)		
<3 years	68	49(27.68%)	19(29.23%)		
Annual family income				1.083	0.582
<50,000	122	92(51.98%)	30(46.15%)		
50∼100,000	77	53(29.94%)	24(36.92%)		
>100,000	43	32(18.08%)	11(16.92%)		
Pre-operative anxiety				10.641	0.002
No	172	136(76.84%)	36(55.38%)		
Yes	70	41(23.16%)	29(44.62%)		
Unstable bladder				2.68	0.102
No	192	145(81.92%)	47(72.31%)		
Yes	50	32(18.08%)	18(27.69%)		
Resected prostate volume				0.194	0.752
<80 mL	169	125(70.62%)	44(67.69%)		
≥80 mL	73	52(29.38%)	21(32.31%)		
Operation time				0.109	0.734
<1 h	186	137(77.4%)	49(75.38%)		
≥1 h	56	40(22.6%)	16(24.62%)		
Catheter retention time				0.677	0.713
1 d	44	34(19.21%)	10(15.38%)		
2 d	55	41(23.16%)	14(21.54%)		
3 d	143	102(57.63%)	41(63.08%)		
Rinse fluid temperature				11.942	0.001
≥20°C	161	129(72.88%)	32(49.23%)		
<20°C	81	48(27.12%)	33(50.77%)		
Rinse liquid speed				7.376	0.009
60∼80 drops/min	163	128(72.32%)	35(53.85%)		
80 drops/min.	79	49(27.68%)	30(46.15%)		
Urinary catheter airbag injection water				10.941	0.002
≤20 mL	191	149(84.18%)	42(64.62%)		
>20 mL	51	28(15.82%)	23(35.38%)		
Blockage of drainage tube				8.926	0.004
No	206	158(89.27%)	48(73.85%)		
Yes	36	19(10.73%)	17(26.15%)		
Post-operative constipation				11.119	0.001
No	163	130(73.45%)	33(50.77%)		
Yes	79	47(26.55%)	32(49.23%)		
Bladder bleeding				5.683	0.021
No	161	110(62.15%)	51(78.46%)		
Yes	81	67(37.85%)	14(21.54%)		

BMI = body mass index.

Diagnostic value of serum PGI2 and 5-HT levels on the occurrence of bladder spasm after TURP surgery

**Figure 2:**
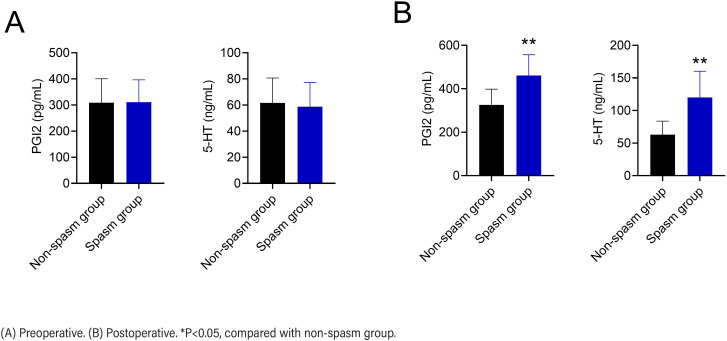
Comparison of serum PGI2 and 5-HT levels in the spasm and non-spasm groups.

**Figure 3:**
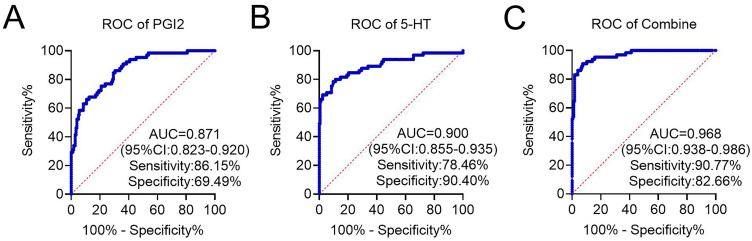
The receiver operating characteristics (ROC) curve and area under the ROC curve (AUC) for predicting the occurrence of postoperative bladder spasm. (A) PGI2. (B) 5-HT. (C) PGI2 combined with 5-HT.

There were no significant differences in preoperative serum PGI2 (310.63±85.07 vs. 309.02±91.41, t=0.128, p=0.898) and 5-HT (89.61±19.55 vs. 58.74±18.38, t=0.312, p=0.756) levels between the spasm and non-spasm groups (Figure-2A). Postoperative serum PGI2 (461.34±95.29 vs. 325.89±72.30, t=11.81, p<0.001) and 5-HT (120.09±40.16 vs. 63.06±20.67, t=14.419, p<0.001) levels were markedly higher in patients in spasm group than in the non-spasm group (Figure-2B). ROC analysis showed that the area under the curve (AUC) for PGI2 in predicting the occurrence of bladder spasm after TURP was 0.871 (0.822-0.911), with a specificity of 86.15% and sensitivity of 69.49% (Figure 3A). The AUC of 5-HT for predicting the occurrence of bladder spasm after TURP was 0.900 (0.855-0.935), with a specificity of 78.46% and a sensitivity of 90.40% (Figure-3B). The AUC for predicting the occurrence of bladder spasm after TURP was 0.968 (0.938-0.986) with a specificity of 90.77% and a sensitivity of 82.66% when the two tests were combined (Figure-3C). The diagnostic efficacy of this combination was better than that of PGI2 or 5-HT alone.

### Univariate and multivariate logistic regression analysis

Postoperative bladder spasms following TURP were found to be associated with a number of factors, including preoperative anxiety, rinse fluid temperature and speed, ureteral balloon injection, obstruction of the drainage tube, postoperative constipation, bladder bleeding, and elevated postoperative serum PGI2 and 5-HT levels (P<0.05, Table-2). With the occurrence of postoperative bladder spasm as the dependent variable, variables that were significant in the univariate analysis were included in multivariate logistic regression analysis (Table-2). The results showed that preoperative anxiety, drainage tube obstruction, and elevated postoperative serum PGI2 and 5-HT levels were independent risk factors for postoperative bladder spasm after TURP (P<0.05).

**Table 2:** Univariate and multivariate analysis of the risk of bladder spasms.

		Univariate			Multivariate	
Indicators	OR	95%CI	P	OR	95%CI	P
Age	0.990	0.965-1.016	0.464			
BMI	1.050	0.992-1.112	0.093			
Education	1.404	0.780-2.527	0.258			
Disease duration ≥ 3 years	1.079	0.576-2.021	0.812			
Annual family income						
50,000∼100,000	1.389	0.736-2.619	0.310			
>100,000	1.054	0.474-2.345	0.897			
Preoperative anxiety	2.672	1.465-4.873	0.001	5.026	1.294-19.521	0.020
Unstable bladder	1.735	0.893-3.373	0.104			
Resected prostate volume	1.147	0.622-2.116	0.660			
Catheter retention time						
1d	1.00					
2d	1.034	0.622∼2.243	0.686			
3d	1.061	0.637∼2.476	0.661			
Operation time ≥ 1 h	1.118	0.575-2.175	0.742			
Rinse fluid temperature < 20°C	2.771	1.539-4.992	0.001			
Rinse liquid speed < 60 or >80 drops/min	2.239	1.243-4.033	0.007			
Urinary catheter air bag injection water > 20 mL	2.945	1.420-6.110	0.004			
Blockage of drainage tube	2.914	1.523-5.578	0.001	7.598	1.919-30.083	0.004
Postoperative constipation	2.682	1.487-4.837	0.001			
Bladder bleeding	0.451	0.232-0.876	0.019			
PGI2	1.020	1.015-1.026	<0.001	1.027	1.017-1.038	<0.001
5-HT	1.071	1.051-1.091	<0.001	1.079	1.050-1.109	<0.001

### Establishment of nomogram model

**Figure 4:**
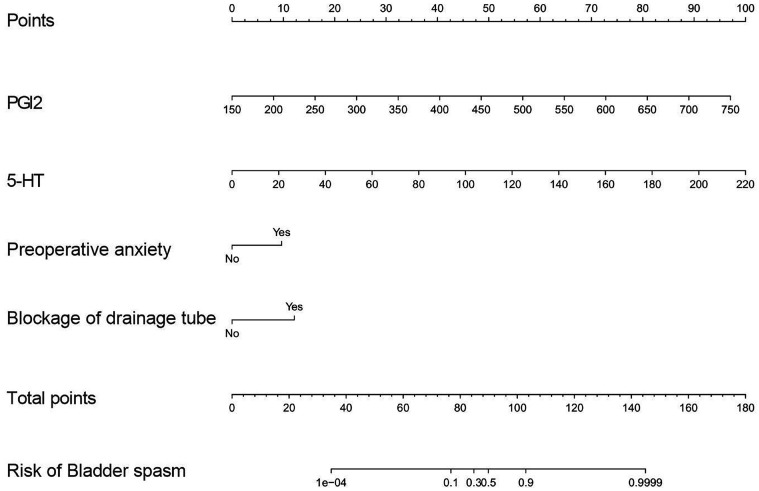
Nomogram based on clinical factors for predicting bladder spasm after TURP in patients with prostatic hyperplasia.

Based on the outcomes of multivariate logistic regression analysis, a nomogram prediction model was created (Figure-4). The degree of influence of each factor on postoperative bladder spasm is presented as a score. The nomogram showed scores of 90.75 for PGI2 level, 100 for 5-HT level, 10 for preoperative anxiety, and 12.5 for drainage tube obstruction, respectively.

### Validation of the nomogram model

**Figure 5:**
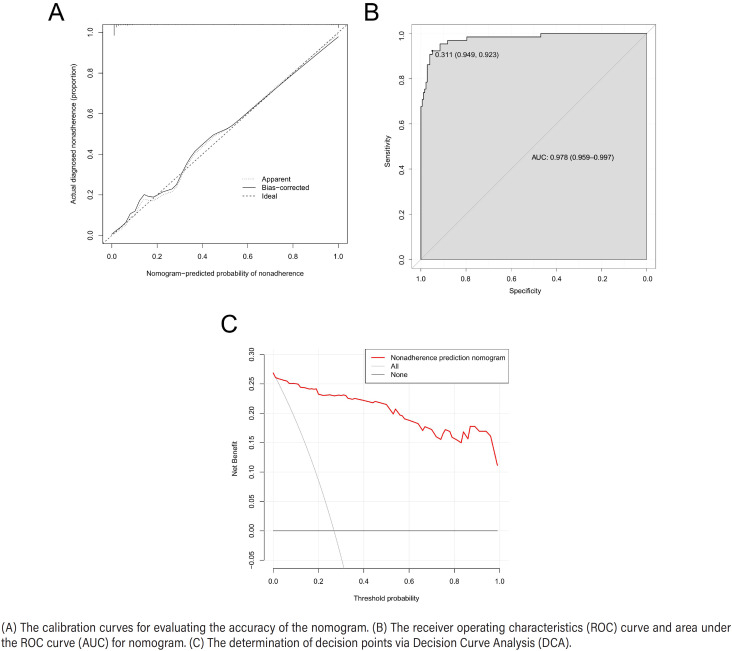
Evaluation of the nomogram model.

The internal validation of the nomogram model was performed using the bootstrap method with 1000 repeated samples. The results showed that the predicted values of the calibration curve of the nomogram model were generally consistent with the actual values (Figure-5A). The C-index of the model was 0.978 (0.959-0.997), suggesting that the model had high diagnostic value. The Hosmer-Lemeshow goodness-of-fit test had χ2 = 4.438, P= 0.816. The ROC curve used to assess the discrimination ability of the nomogram model showed an AUC of 0.978 (0.959-0.997) (Figure-5B). The clinical diagnostic value of the model was validated using DCA (Figure-5C). The net benefit of using the nomogram to predict the risk of postoperative bladder spasm was greater when the predictive value of the nomogram model ranged from 0.1–1.0, indicating that the model had a high assessment value.

## DISCUSSION

Patients with prostate enlargement frequently experience bladder spasms after surgery. These conditions can impair wound healing, induce paroxysmal suprapubic discomfort, and potentially result in secondary bleeding and urinary tract infections, all of which can hinder recovery (13). Previous studies have shown that the incidence of bladder spasm after TURP ranges from 15.79% to 55.71% (14). The present study showed that the incidence of post-TURP bladder spasm in patients with prostate enlargement was 26.86%, consistent with previous reports. Important to cite that bladder spasms, or postoperative pelvic pain, cause is still unclear, and the symptoms may last longer in about 15% of the cases, influencing the patients’ quality of life (15). It is clinically important to identify risk factors associated with the occurrence of postoperative bladder spasms.

Several causative factors can lead to bladder spasms. In this study, we found that preoperative anxiety, rinse fluid temperature, rinse fluid speed, urethral balloon injection, drainage tube blockage, postoperative constipation, and bladder bleeding were associated with the development of bladder spasms after TURP. Emotions such as anxiety and fear can decrease sympathetic tone, and detrusors are subjected to inhibitory effects from the pituitary-hypothalamic-adrenocortical system, leading to decreased bladder stability (16). Furthermore, poor extraction and bleeding from the indwelling line are likely to result from increased production of bradykinin and other chemicals, which raises the risk of bladder spasm. For postoperative bladder care, the temperature and rinse rate during bladder irrigation are crucial (14, 17). Low rinse fluid temperature not only causes irritation in bladder smooth muscle but also causes hypothermia in patients. Moreover, a low rinse fluid temperature may lead to decreased prothrombin activity and aggregation of coagulation factors in the body, thereby increasing the risk of catheter blockage. Therefore, intraoperative bladder rinse fluid should be preheated to approximately 30°C before use and the patient's bladder status should be monitored during the rinse procedure. In this study, bladder irrigation fluid during bladder irrigation was injected at a rate of 60–80 drops/min. A slower speed leads to a poor flushing effect and may cause poor drainage and increased pressure in the bladder. In contrast, a fast bladder irrigation speed can affect smooth muscle contraction and stimulate detrusor while elevating bladder sympathetic excitability (18). Excessive filling of the urethral balloon increases the pressure in the bladder neck and urethra, which in turn stimulates the bladder sensory nerves and causes bladder contraction. Wilde et al. (19) showed that drainage tube blockage can cause catheter-associated urinary tract infections, and urinary tract which can produce inflammatory stimulation of the bladder triangle, in turn inducing bladder spasm. Therefore, it is clinically necessary to ensure that the drainage tube is patent to avoid tortuosity, folding, and blockage. In addition, alterations in the ultrastructure of the hypocompliant or unstable bladder result in electro-coupled depolarization between detrusor cells. As a result, detrusors are prone to persistent contractions during urination, which in turn induces bladder spasm (20, 21). However, this study did not find a difference in the proportion of patients with unstable bladders between the spasm and non-spasm groups, which may be related to the small sample size.

PGI2 is produced by cyclooxygenase-2 (COX-2) and plays an important role in the regulation of the lower urinary tract function. Dilation during detrusor filling, bladder inflammation, mucosal injury, and muscarinic receptor stimulation can induce PGI2 expression in bladder tissue (22). By stimulating capsaicin-sensitive afferent neurons to reduce the stimulus threshold necessary to cause bladder contractions, overexpression of PGI2 contributes to the voiding reflex. Urinary symptoms associated with bladder outlet blockage and/or intravesical alterations can cause the bladder's PGI2 to rise. This elevated PGI2 then contributes to the development of symptoms related to the lower urinary tract, including urgency, frequency, and incontinence. Therefore, patients with irritative bladder symptoms such as urinary frequency and urgency can be assessed for lower urinary tract function through alterations in PGI2 release (23).

Neurotransmitter and vasoactive compound 5-HT is extensively diffused in the central nervous system and peripheral tissues. It has been demonstrated that the voiding regulation center, which is housed in the hypothalamus, regulates the lower urinary tract by combining the actions of many neurotransmitters, including 5-HT (24). Andersson et al. (25) demonstrated that 5-HT levels in the brain are directly related to lower urinary tract function. Numerous 5-HT-containing downstream neurons innervated to the spinal nucleus are received by the autonomic nucleus of the lumbosacral segment along with the somatic nucleus. Multiple 5-HT receptors are dispersed at locations that process afferent and efferent impulses to the lower urinary tract. 5-HT can either cause or prevent voiding, depending on these receptors (26). Overactive bladder illness has been treated with 5-HT reuptake inhibitors as an adjuvant (10). Alterations in 5-HT levels have been associated with the development of depression and anxiety (27), and psychiatric disorders such as depression and anxiety both trigger the development of postoperative bladder spasms (28). In this study, we found that the postoperative serum PGI2 and 5-HT levels were significantly higher in patients with bladder spasms than in those without spasms. In addition, the predictive value of both was higher for the occurrence of postoperative bladder spasms, and their diagnostic efficacy was higher when combined than when used alone. Therefore, serum PGI2 and 5-HT levels can be used as noninvasive diagnostic markers for diagnosing postoperative bladder spasm after TURP.

The nomogram approach allows healthcare practitioners to more easily forecast bad events on an individual basis by presenting the risk variables filtered by logistic regression analysis as scores. This allows for a visual assessment of each risk factor's contribution to adverse events (29, 30). Plotting nomograms using publicly accessible clinical data and blood biochemical markers is a commonly utilized method for risk assessment of adverse event occurrence (31, 32). For instance, a nomogram model that predicts a poor prognosis for patients receiving radical cystectomy for uroepithelial carcinoma may be created by combining systemic inflammatory response indicators with clinicopathological data (33). Nevertheless, there are currently no published prediction algorithms that accurately forecast the likelihood of bladder spasms following TURP. In this study, we found that preoperative anxiety, drainage tube occlusion, and elevated postoperative serum PGI2 and 5-HT levels are independent risk factors for the development of postoperative bladder spasm after TURP. Importantly, the nomogram model developed based on serum PGI2 and 5-HT levels and the independent risk factors affecting postoperative bladder spasm after TURP showed good precision and discrimination. When the established prediction model was validated, it was found to have good efficacy in terms of the prediction accuracy, calibration curve, and DCA. Therefore, the nomogram model constructed in this study is a valuable tool for assessing postoperative bladder spasm. Additionally, bladder spasm is a difficult to treat condition. Maybe identifying individuals prone to develop this condition may trigger early treatment. Nevertheless, treatment still needs to be further evaluated and the urological Community treats this situation in different manners (15).

To the best of our knowledge, this study was the first to construct a mathematical model for predicting the occurrence of bladder spasms based on humoral biomarkers versus clinical parameters and showed additional clinical benefit. However, this study has several limitations. First, this was a single-center study with a small sample size and a limited population size, reflecting clinical characteristics. Second, as a retrospective analysis, the study population was not randomly included, and the case screening process was subjectively biased, which may have influenced the results. Third, the nomogram model was only used for internal validation, and it could not be determined whether the extrapolation of the model was good. Moreover, as a retrospective analysis, this study used very strict inclusion and exclusion criteria, which to some extent ensured a high degree of consistency in the characteristics of the population and the accuracy of the model, but this advantage also limited the scope of use of the model. The involuntary contraction of the bladder detrusor muscle is regulated by acetylcholine M-type receptors. Study has shown that before awakening from anesthesia after surgery, 2% lidocaine 10 mL and atropine 0.5 mg are injected through the catheter. Lidocaine reduces the sensitivity of the bladder mucosa through local anesthesia, and atropine can specifically block the transmission of acetylcholine in the nerve endings, and the combination of these two drugs can safely and effectively reduce the incidence of bladder spasm after awakening from anesthesia (34). In terms of postoperative medication, although anticholinergic drugs can effectively relieve the symptoms of bladder spasm, but the systemic adverse effects are obvious, while non-anticholinergic antispasmodic drugs such as resorcinol can prevent postoperative bladder spasm and at the same time reduce the anticholinergic-like adverse effects, such as dry mouth and dry eyes (35). The samples included in this study were all operated on by the same surgeon who performed the relevant procedures with the same anesthesia regimen. In addition, in the postoperative period, the patients all received the same epidural self-controlled analgesia. Therefore, these factors in the perioperative period may not influence our conclusions. Of course, the model constructed in this study does require subsequent prospective studies, which is the direction of our future research.

In conclusion, preoperative anxiety, drainage tube occlusion, and elevated postoperative serum PGI2 and 5-HT levels were independent risk factors for the development of bladder spasm in patients with prostate enlargement. The nomogram prediction model based on the risk factors affecting bladder spasm after TURP was helpful for the risk assessment of bladder spasm occurrence and had a guiding value in avoiding or reducing post-TURP complications.

## COMPLIANCE WITH ETHICAL STANDARDS

This study was approved by The Ethics Committee of Heji Hospital Affiliated to Changzhi Medical College (2020-CZ-006). Informed consent was obtained from the participants for participation in the study, and all methods were performed in accordance with relevant guidelines and regulations.
